# Testimony ceremonies in Asia: Integrating spirituality in testimonial therapy for torture survivors in India, Sri Lanka, Cambodia, and the Philippines

**DOI:** 10.1177/1363461512447138

**Published:** 2012-07

**Authors:** Inger Agger, Victor Igreja, Rachel Kiehle, Peter Polatin

**Affiliations:** Rehabilitation and Research Centre for Torture Victims; University of Queensland; St. Mary’s Hospital, Amsterdam, New York; Rehabilitation and Research Centre for Torture Victims

**Keywords:** torture, trauma, Asia, transcultural healing methods, ceremonies, spirituality

## Abstract

This study explores the therapeutic implications of including culturally adapted spiritual ceremonies in the process of testimonial therapy for torture survivors in India, Sri Lanka, Cambodia, and the Philippines. Data were collected through an action research process with Asian mental health and human rights organizations, during which the testimonial method was reconceptualized and modified to include four sessions. In the first two sessions, community workers assist survivors in the writing of their testimony, which is their narrative about the human rights violations they have suffered. In the third session, survivors participate in an honour ceremony in which they are presented with their testimony documents. In the fourth session, the community workers meet with the survivors for a reevaluation of their well-being. The honour ceremonies developed during the action research process came to employ different kinds of symbolic language at each site: human rights (India), religious/Catholic (Sri Lanka), religious/Buddhist (Cambodia), and religious/Moslem (Philippines). They all used embodied spirituality in various forms, incorporating singing, dancing, and religious purification rituals in a collective gathering. We suggest that these types of ceremonies may facilitate an individual’s capacity to contain and integrate traumatic memories, promote restorative self-awareness, and engage community support. Additional research is needed to determine the method’s applicability in other sociopolitical contexts governed by more Western-oriented medical traditions.

Testimonial therapy is an individual psychotherapy method for survivors of human rights violations. It is a brief psycho-legal approach to trauma, which involves the narration of survivors’ traumatic experiences. The testimony method was derived in more Eurocentric settings. However, during the action research process conducted with the Asian organizations, the method was reconceptualized and modified, taking into account Asian cultural and spiritual dimensions. The present study aimed to explore the therapeutic impact of including culturally and religiously adapted ceremonies within testimonial therapy with torture survivors.

The study is based on action research with five Asian mental health and human rights organizations^1^. The action research framework included eight testimonial therapy workshops held in India (five), Sri Lanka (one), Cambodia (one), and the Philippines (one) from May 2008 through April 2010, during which the testimonial therapy method was introduced by the Rehabilitation and Research Center for Torture Victims (RCT) and further developed in cooperation with the Asian partners. The results presented in this paper are based on an analysis of the data collected during the eight workshops, and expand on previous research on testimonial therapy conducted in India (Agger, Raghuvanshi, Khan, Polatin, & Laursen, 2009).

In South and Southeast Asia, there are few resources for the provision of therapeutic assistance to torture survivors, and it was the intention of RCT to conduct a capacity-building program for Asian nongovernmental organizations (NGOs) by means of workshop training in testimonial therapy, which constitutes a brief psycho-legal approach to trauma. The action research described in this article evolved as a result of the interest and engagement shown during the first pilot workshop in India with the People’s Vigilance Committee on Human Rights (PVCHR) in May and June 2008 and subsequently by the members of the other organizations who participated in the workshops. There were two phases to this process: (a) Interactive participation in the course of the workshop process during which the workshop trainer (Inger Agger) recorded her observations in detailed field notes, conducted interviews with survivors and NGO staff about their experience, collected data on local healing practices, and constructed hypotheses to further develop the testimonial therapy method. (b) Clarification and analysis of the qualitative data by the trainer in cooperation with the coauthors. The results of this reflective process are presented here.

In the following, we will first briefly discuss the previous use and subsequent evolution of testimonial therapy in different geographical and cultural settings, and describe some Asian healing practices and their relation to newer Western methods. The concept of “interoception” will be introduced as a theoretical framework for understanding the therapeutic qualities of the spiritual practices presented in this study. We will then outline the action research method, which was developed in the course of the workshop process during which the data for this study was collected. We will present the common elements of the testimony ceremonies created in the action research process, and illustrate them with case examples in four different cultural, religious, and geographical contexts. We will end with a discussion of the implications and significance of the reconceptualization and modification of the testimonial method.

## Development of testimonial therapy

In 1983, two Chilean therapists (Cienfuegos & Monelli, 1983) writing under pseudonyms presented and analysed testimony as a specific therapeutic technique which was being used to confirm and document severe emotional trauma that victims of torture and their relatives had experienced. Since then, the method has been used with modifications to help survivors of violence-related trauma in different regions of the world (Agger, 1994; Agger & Jensen, 1990, 1996; Akinyela, 2005; Curling, 2005; De la Rey & Owens, 1998; Igreja, Kleijn, Schreuder, van Dijk, & Verschuur, 2004; Luebben, 2003; Lustig, Weine, Saxe, & Beardslee, 2004; Neuner, Schauer, Klaschik, Karunakara, & Elbert, 2004; Neuner, Schauer, Roth, & Elbert, 2002; van der Veer, 1992; van Dijk, Schotrop, & Spinhoven, 2003; Weine, Kulenovic, Pavkovic, & Gibbons, 1998). Testimony is also applied as part of narrative exposure therapy, which has been manualized for use in contexts of war and conflict (Schauer, Neuner, & Elbert, 2005). In a recent randomized, controlled trial with 26 torture survivors in Sri Lanka, Puvimanasinghe and Price (2010) found that testimonial therapy significantly improved psychosocial functioning and concluded that this method seems to be “a viable therapy for torture survivors collectively seeking justice” (2010, p. 4).

Testimony is a narrative about an event: a trauma story told by a witness who suffered an injustice or something painful or terrible. In some cultures, the witness can even assume the spirit of a victim of traumatic death that purposefully possesses a living person for truth telling (Igreja & Dias-Lambranca, 2008). The story can be narrated in words or interpretive performance using elements of music and art. The testimony may have objective functions: as evidence, attestation, proof, or advocacy; and it may also function as a subjective expression of disapproval, condemnation, or protest. Testimony giving can become an occasion for cultural expression and affirmation. It may also express individual creativity in mediating memories of violent experiences (Igreja, 2010; Krog, Mpolweni, & Ratele, 2009).

Mollica (2009) has underlined the importance of helping the survivor to create a narrative about the traumatic events that contains healing metaphors so that bearing witness does not become “toxic” for the person narrating the story and for people listening to it. The primary goal is to transform the trauma story from an account of shame and humiliation into one of dignity and virtue. The audience for the testimony can include respected persons in the communities (e.g., village head) and persons significant to the survivor (e.g., family).

## Asian healing practices

“Embodied spirituality” conceives of body and mind as a unit. This concept is an important element of daily life of Asian cultural contexts in which our research took place. In India many aspects of religious and cultural practices relate to the healing process. The ancient Vedic system of healing, Ayurveda, establishes a relationship between mind, tolerance, memory, and meditation in seeking the causation and healing of trauma (P. Upadhyaya, personal communication, December 12, 2009; see also Cornelissen, Misra, & Varma, 2011). Somasundaram (2007) has emphasized the importance of cultural rituals and ceremonies to address the many problems of survivors following exposure to conflict, war, and disaster in Sri Lanka. In Cambodia, family and community support, along with Buddhist religious practice—taking part in chanting, receiving blessings from monks, wearing amulets and magic bands—are important for healing of *Baksbat*, which is a culturally tied syndrome of worries, unhappiness, pain, and distress (S. Chhim, personal communication, October 7, 2009; Hem, 1996). In Philippine healing traditions, a Catholic priest, an *ustadz* (Muslim teacher), or a *babaylan* (female indigenous healer) may give blessings and pray for an individual who is affected by physical or mental illness. *Babaylans* among the indigenous tribes on the island of Mindanao perform rituals to bring a person back to health. The ritual is normally conducted on regular worship days inside a *ventana* (sacred worship place), and the *babaylan* is usually in a trance state whenever she conducts healing sessions. Healing processes are often linked to supernatural powers or the “unseen” (i.e., *Usog*, *bati*, or *engkanto*), and numerous people in rural areas still believe in “seers” and consult them (J. A. Lascano, personal communication, April 20, 2010).

The inclusion of culturally adapted spirituality in the therapeutic process therefore appears to be an approach that may relate to the inner world of many Asian survivors of torture. Interestingly, Asian spiritual practices have already become an important element of the Western “third wave” of cognitive–behaviour therapy represented by Acceptance and Commitment Therapy (ACT) (e.g., Hayes & Smith, 2005), and Dialectical Behaviour Therapy (DBT) (e.g., Linehan, 1995). Mindfulness-based stress reduction (MBSR) (e.g., Didonna, 2009) and mindfulness-based cognitive therapy (MBCT) (Segal, Williams, & Teasdale, 2002) are inspired by Buddhist meditation and yoga, and have proved effective for prevention of depression as well as narrative integration in a Western context (Kabat-Zinn, 1994). With globalization, healing approaches move around and across religious, cultural, and political boundaries.

## Interoception, embodied spirituality and healing

Interoception is the self-observation of internal sensations, images, and affects in the here and now, and appears to be an important means to promote healing after psychological trauma (Levine, 2010; Ogden, 2009; Porges, 1995; Siegel, 2010; van der Kolk, 2006). The main challenge, however, is to engage in the observation of disturbing sensations, images, and feelings while simultaneously regulating the automatic defense mechanisms of fight, flight, and freeze in which the individual seeks safety and control. Such defensive responses may result in dissociation from the immediate world, which prohibits the integration process.

We propose that there are basic psychological elements in different cultural healing practices that relate to the interoceptive capacities for observation of internal sensations and the regulation of physical arousal. Traumatizing events with their accompanying images, sensations, and feelings may become autonomous fragments in the mind (Frewen et al., 2008; Hopper, Frewen, van der Kolk, & Lanius, 2007; van der Hart & Steele, 2000; van der Kolk et al., 1996; van der Kolk, Roth, Pelcovitz, Sunday, & Spinazzola, 2005). These fragments may distract the affected individual from engaging in new experiences that could lead to resolution and healing because he or she is preoccupied with goals of *not feeling*, *thinking of*, or *experiencing* these traumatic memories. Trauma survivors may be able to describe their experiences objectively, but cannot necessarily address the vital personal issues of helplessness and guilt. They are commonly locked into a cycle in which they reexperience and then attempt to avoid their traumatic memories. This can lead to a loss of one’s sense of purposeful identity and to an alienation from others.

Mindfulness, yoga, and spiritual practices serve to engage observational capacities while regulating physiological arousal by means of activities such as breath work, movement, and reverential practices. Spiritual rituals may generate a sense of being part of a larger context, thereby allowing disturbing feelings and memories to be confronted. Spiritual practices, along with physiological tracking, can be integrated in the testimonial work, and make it easier for the individual to face disturbing images. It may serve to hold together the fragmented psyche so as to enable awareness of the present moment. This process may allow integration of sensations, images, and feelings within the narrative (Le Doux, 2000; Perry, 1999). However, unless the body can tolerate all the sensations associated with the trauma, testimonies tend to be repetitive, cliché-ridden narratives that may not capture the essence of the event and are likely to omit the most salient details. Addressing this issue proved to be one of the major challenges in the testimony workshops.

## Method

The aim of the first workshop in testimonial therapy with the Indian NGO PVCHR in May and June 2008 was to explore the usefulness of testimonial therapy for mental health and human rights organizations in Asia. During this pilot project, the workshop trainer and PVCHR staff started to raise questions about the applicability in an Indian context of the testimonial method as it had previously been applied in other cultural settings. During the first workshop, the process of solving these problems and the knowledge gained through teamwork converted the workshop into an experience that can best be described as “participatory action research” (Whyte, 1991). Thus, the results of this pilot workshop were so promising that the trainer decided to pursue the action research approach in the following seven workshops each of which lasted 2 weeks and were conducted from 2008 through April 2010. During this workshop process, the testimonial therapy method was progressively developed and modified in response to local challenges and problems. Thus, the workshop trainer became a participant observer of the process, which was investigated and documented by the trainer in field notes, training material, interviews, reports, videos, and conference presentations.

Action research has a long tradition, building amongst others on the critical pedagogical work of Freire (1970) in Latin America. Dick (2002, p. 2) defines action research as: “A flexible spiral process, which allows action (change, improvement) and research (understanding, knowledge) to be achieved at the same time”. The trainees from the Asian NGOs participating in the workshops became truly engaged in changing the testimonial therapy model and achieving new knowledge about how best to support torture survivors in a developing country with few resources for provision of therapeutic assistance. The trainee groups were primarily human rights activists and community workers. Some had no prior mental health training. The trainer always had a local staff member as her cotrainer who could later train other staff or NGOs.

The first week of the workshop focused on theory about testimonial therapy and mental health issues among torture survivors, as well as practical exercises during which trainees were involved in role-playing. Trainees practiced with each other how to take testimonies, playing in turn the role of: the *interviewer*, who interviews the survivor and elicits his or her narrative about the torture; the *note-taker*, who takes notes during the interview and writes the narrative of the survivor; and the *survivor*. The trainees read their testimonies out loud and the trainer, cotrainer and other trainees gave feedback about the writing style and the type of narrative engendered, looking into whether the testimony focused heavily on legal issues, included psychological issues, coping and positive elements, or only focused on pain.

During the second week of the workshop, each trainee was supervised as they took one to two testimonies with survivors at the training venue. Transport and accommodation were arranged for the survivors when necessary. The local NGO in cooperation with the trainer and cotrainer selected survivors who exhibited signs of psychological distress. Most of the survivors had previously sought legal assistance or counseling. The NGOs involved made sure that survivors were fully informed about the public testimonial ceremony and knew their right to decline without penalty.

After the first two sessions with the survivors, the NGO staff transformed the narratives into a visually attractive document. In the meantime, the trainees in cooperation with the trainer and cotrainer started planning the location and elements of the ceremony wherein the testimony documents would be presented to the survivors. They incorporated local healing traditions and honouring practices from each country. As a participant observer, the trainer kept notes on the ritual elements of each ceremony, and interviewed NGO staff about local terms and symbolic meanings of ceremonial elements.

The trainer participated actively in the ceremonies. She was normally given a role in the ceremony, such as handing testimony documents or flower garlands to survivors. After the ceremonies, the workshop trainer and cotrainer met with survivors and trainees to evaluate their experience of the event. Interviews were in an unstructured format beginning with the question: “How did you experience the ceremony?” In most cases, the trainer and cotrainer first met with the survivors and asked them to describe their experience during the ceremony. Trainees were subsequently asked about their perceptions of the ceremony.

Once the workshop process had been concluded in April 2010, the workshop trainer presented her preliminary findings from the action research process at an international conference^2^. The trainer then invited the three coauthors of this article, to review the preliminary data and collaborate with her on the analysis presented in this article.

## Results: Ceremonies in four cultural contexts

The testimonial method, which was developed during the action research process, came to include four sessions^3^: The first two sessions involve a survivor, an interviewer, and a note-taker. During Session 1, the trauma story is “opened” by the survivor and the interviewer, and the first draft of the testimony is written by the note-taker in cooperation with the interviewer after the session. In Session 2 the narrative is “closed,” and the survivor, interviewer and note-taker arrive at a written statement for the survivor to approve. Session 3 consists of the testimony ceremony, which honours the survivor and can be adapted to the various contexts, as demonstrated by the examples described in what follows. In Session 4, one to two months after the ceremony, the interviewer meets with the survivor to assess his or her well-being.

The survivors had experienced different forms of political violence and torture, which led to their distress. In India, Sri Lanka, and the Philippines, the survivors had been tortured physically and/or mentally primarily by present-day government agents, while the survivors who were part of the workshop process in Cambodia had experienced crimes perpetrated by the Khmer Rouge regime from 1975 to 1979.

A total of 245 survivors participated in 43 ceremonies during the period covered by this study (2008–2010): in India 121 survivors participated in 11 ceremonies; in Sri Lanka 31 survivors in 21 ceremonies; in Cambodia 53 survivors in 6 ceremonies; and in the Philippines 40 survivors in 5 ceremonies.

The ceremonies reflected the different cultural, political, and spiritual approaches to healing. As the action research turned out, eight common ritual elements could be identified:

- The ceremony is performed at a symbolic *location*, such as a sacred space, a place of remembrance, or a place representing oppressive forces.
- A few key *actors* perform the ritual elements of the ceremony. This may include representatives of religious or local communities, or other respected persons who can give the ritual a higher significance.
- An *audience* of significance to the survivor is present. It can include members of the community, family, friends, colleagues, and the media.
- There is a visually impressive *document* containing the narrative to reflect that the “bad” story has been symbolically reframed by inscribing it on fine, nicely bound paper.
- A community worker reads out the document in the first person, so that the survivor gets an *auditory* impression of his or her story, thus facilitating mindfulness and acceptance by hearing the anxiety-provoking story in another person’s “safe” voice.
- The document is *presented* to the survivor by someone of importance together with other *symbols of honour* such as flowers, shawls, protective bands, etc. The story is “given back” to the survivor to keep in its new and positive version, which the survivor can use as he or she wishes for advocacy, remembrance, or family history.
- The document is symbolically *purified* by one of the key actors in the ceremony: The priest, imam, or monk says a prayer over the document, or the human rights leader gives a speech honouring the struggle of the survivor.
- Interventions *embodied with spiritual significance* are performed, such as singing, dancing, chanting, hugging, touching, and *sharing of a meal*. Emotional release is facilitated through expressions of love, kindness, and compassion.

Examples from each country are described below.

### India

A total of five workshops on testimony therapy were held in India for different human rights organizations in 2008 and 2009, during which 75 community workers and human rights activists were trained. The first manual of the testimony method was produced (Raghuvanshi & Agger, 2008) in cooperation with the PVCHR in Varanasi during the pilot workshop in May–June 2008. Following this workshop, the PVCHR held 10 additional testimony ceremonies within the research period in the form of: (a) public demonstrations in front of government headquarters, (b) “Peoples’ Tribunals,” that is, hearings bringing attention to critical human rights issues, (c) community meetings in villages, (d) “Folk School”—popular schools for the poor and marginalized—meetings, and (e) street plays and singing events. Group discussions with selected survivors and PVCHR staff were conducted in December 2010 during which the testimonial method was discussed and evaluated by survivors and staff. An example of a ceremony in a village community is described in what follows.

#### Village community ceremony

The ceremony was organized for five survivors, two women and three men, who had suffered police torture. The ceremony was held in a symbolic location, the village of Raup in which people had previously been victimized by local police. The Ghasia tribe, an indigenous group of 79 families, live in Raup. The PVCHR has used this location for several honour ceremonies because Raup symbolizes a “success story” for tribal people. In 2000, the Ghasia tribe engaged in an oppositional effort against police exploitation. They consequently suffered police brutality and destruction of resources leading to widespread malnutrition and the deaths of 18 children. In the last few years, they have significantly restored their living conditions with the help of the PVCHR and erected a memorial for those who died (Raghuvanshi, 2011).

The “guests of honour” and key actors leading the ceremony were the director of the PVCHR who gave an introductory speech, a local dignitary from the municipal government, the workshop trainer, and the human rights workers that had taken the testimonies from the survivors. Also present were representatives of three local newspapers. The audience consisted of Raup villagers and survivors who had been honoured in previous ceremonies. In addition, around 300 people from 10 surrounding villages attended the ceremony.

The program started with a “*Karma dance*” in honour of the holy *Karma Tree*, which stands for fortune and good luck. Two villagers played the drums, and the villagers sang two “testimonial” songs that they often use to express their suffering. The first song was about their living conditions and the second song a narrative of their memories of collective traumatic losses:

So miserable has been our life since birth

Leaving our native places we have to

Live away, away in hills

So unfortunate our lives have been

Others enjoy delicious lentils, boiled rice

The aborigines are destined to eat coarse *Kodo*

So unhappy forlorn has been our life!

Displaced, discarded, we are forced to live in the hills.

The villagers appeared visibly moved by this performance as evidenced by their attentive body posture and sad facial expressions. The human rights workers then read out a summary of the five survivors’ testimonies and gave the documents to the survivors who were also presented with the traditional white shawl and a flower garland, symbols of purity and honour. PVCHR staff took photographs while the testimony documents were given to the survivors by one of the key persons, and survivors were also photographed holding their testimony documents. The program ended with a vote of thanks and homage to the monument to the children. The next day, local newspapers published stories about the event, thus reinforcing the sense of collective and official recognition of the villagers’ suffering.

The trainer held a group discussion with the survivors some months after the ceremony in which they said that they no longer felt as fearful as before the therapy, and that they felt more connected to their families and community. They also felt that their dignity had been restored. One of them added that, as a result of the therapy, she had started to support and counsel other survivors. Another survivor believed that he had become “irritating” to the authorities because he no longer behaved as submissively as expected towards the police. Many verbalized a reduction of phobic symptoms.

A discussion group for the NGO staff was held on the same occasion. They emphasized that survivors who had completed testimonial therapy had made positive change in social functioning, increased their capacity to support others and had become less distressed. They also underlined the importance of preparing survivors for public exposure, which he or she would experience during the ceremony.

### Sri Lanka

A testimony workshop was held in Sri Lanka in October 2008 for members of the human rights network “People Against Torture.” Twenty-two psychologists, community workers, and human rights activists participated in the workshop. During the workshop process, the manual for India was revised for the Sri Lankan context and published in December 2008 (Perera, Puvimanasinghe, & Agger, 2008). Revisions included an introduction on the human rights situation in Sri Lanka and the rephrasing of certain sentences so that they would became more “neutral” in view of the tense political situation in the country at that time. At the end of the workshop, two NGOs conducted testimony ceremonies, in both of which the workshop trainer took part as a participant observer.

During the period following the workshop until April 2010, 31 torture survivors participated in testimonial therapy. A total of 23 ceremonies were held for the survivors: 8 were large public human rights events with more than 50 people; 1 was a small public human rights event in a village; 5 were held as a part of victim support group meetings; 1 was a private event for family, friends, and neighbours; 1 was a Buddhist meeting; 1 was a Muslim meeting; and 6 were Catholic–Hindu meetings. Here is an example:

#### A Catholic–Hindu ceremony

The ceremony was held for a Tamil survivor of police torture. This man had been imprisoned on suspicion of being a Tamil Tiger^4^. The ceremony took place at a Catholic mission station where the director of the human rights organization “Home for Victims of Torture” (HVT) lived and worked as a parish priest. The mission station was viewed as a sacred space and a place of refuge by a number of both Catholic and Tamil Hindu persecuted people. The key people leading and performing the ceremony were the priest who was dressed in white—a colour symbolizing purity—and HVT staff members. The audience consisted of the survivor’s Tamil family and members of the HVT support group. This group included mostly middle-class, Catholic representatives of the local community. The survivor and his family were seated in front of the audience.

The ceremony started with a welcome speech by an HVT staff, who invited the audience to stand up and applaud the survivor and his family “to welcome them into our ceremonial community.” After the speech, two girls dressed in white prostrated themselves at the feet of the survivor and his family and presented them with betel leaves, which are a symbol of honour. Another HVT staff member led the audience in a meditation:

We relax our body. We become aware of our breathing pattern, of our inhaling and exhaling. When silence is prevailing within and around us, we will listen to a wise song. The song speaks of the human dignity and human respect all of us inherited at birth. We are all human beings, born into one human family. It is our birthright. Such thoughts indeed prepare us for this great occasion.

A traditional Sri Lankan song, *Jaya Mangala Gatha* invoked blessings and good wishes. Then, the survivor and his family, the priest, and the main guests of honour were asked to light lamps as a special sign of honour. An HVT staff member declared: “The symbol in front of us shows how the degraded human dignity of this family is rebuilt and upheld again, how the social connections are revived, how life once under trauma is being healed.” Three girls performed a traditional *Sinhala* dance, and two others performed a traditional *Pooja* dance to invite the gods to the occasion.

An HVT staff member then read out the testimony, after which the testimony *document* was presented on a tray covered with a white cloth to the survivor by another survivor who had also experienced imprisonment and torture and had gone through a counselling process with HVT. He paid tribute to the bravery and struggle of the survivor, and gave him words of encouragement drawing parallels with own personal experience. The priest thanked the participants and also paid tribute to the bravery of the survivor.

At the end of the ceremony, the audience expressed their best wishes and encouragement to the survivor and his family: One by one they hugged the survivor. The survivor, his family, and the audience were moved to tearfulness. Afterwards, a common meal was shared and a video on human rights shown. HVT had decided also to use the ceremony for advocacy and to raise awareness of human rights.

In an interview after the ceremony, the survivor said that he felt “great” because he had been able to share his pain with a group who had shown him affection and solidarity. The staff noted that a testimony ceremony could facilitate social reintegration of survivors who have often been marginalized in their communities because they were suspected of being “terrorists.” The staff also remarked that a testimony ceremony could serve as an important closure of a longer term counseling process.

### Cambodia

A testimonial therapy workshop was held in Cambodia in October 2009 for 18 staff members (psychologists, psychiatrists, and counselors) of the Transcultural Psychosocial Organization – Cambodia (TPO), an NGO. As part of the training, testimonies were taken from 16 survivors of the Khmer Rouge regime. At the end of the workshop, the trainer, cotrainer, and trainees organized a testimony ceremony during which the survivors were honoured. The survivors were all “civil party” applicants^5^ in the first case of the Khmer Rouge Tribunal (officially named the Extraordinary Chambers in the Courts of Cambodia – ECCC) against Duch, the former head of the torture centre known as *Tuol Sleng* or S-21. The hearing had ended in September 2009, just before the testimonial workshop was held. Survivors had expectations of obtaining justice through this tribunal^6^. Although silence or forgetting are often conceptualized as the proper response to traumatic events in a Buddhist context, the fact that the Khmer Rouge Tribunal was taking place had encouraged some survivors to narrate their stories so that the perpetrators could be brought to justice. After the workshop, the TPO conducted five additional testimony ceremonies for a total of 37 survivors.

The following is a description of the Buddhist testimony ceremony developed during the workshop. The workshop trainer took part in the ceremony as a participant observer.

#### A Buddhist ceremony

The ceremony was held outside of Phnom Penh for 16 civil party applicants. They were all survivors of Khmer Rouge atrocities. Most of them had been imprisoned and tortured, while others had lost close family members. A film crew from the Cambodian NGO Bophana Audiovisual Resource Center joined the ceremony to record the event. Later, Bophana produced a 16-minute long documentary on the ceremony (Priour, 2010), which hopefully will serve as a tool for advocacy for survivors’ rights to justice.

The ceremony took the form of a collective “pilgrimage” to two different symbolic locations. The participants first travelled 15 kilometers by bus from Phnom Penh, the capital city, to *Choeung Ek* also referred to as the “Killing Fields,” where 17,000 people are believed to have been executed during the Khmer Rouge regime from 1975 to 1979. This first location was a symbolic place of remembrance with a Buddhist *Stupa* (religious monument),containing human skulls of more than 5,000 victims. The second part of the pilgrimage took place at the *Sala chhan*, or place for public functions, of a nearby pagoda. This location was chosen because it represented a sacred space with a spiritual and soothing atmosphere. The key individuals taking part in the ceremony were three Buddhist monks, a lay person, *Achar*, who led the ceremony, and the TPO director and counselors. The audience consisted of 25 other TPO staff members.

In the first ceremonial location*,* survivors proceeded to walk through the “Killing Fields” reminiscing about their experiences during the Khmer Rouge period. The large number of graves and residual bones prominently displayed at that location brought back painful memories accompanied by sorrow and anger as observed by the workshop trainer. Survivors highlighted areas of interest to the other participants in the ceremony. The ceremony then started with a *Bang Skool* ritual held in front of the *Stupa*. This was a symbolic offering to the ancestors made through the monks, addressing people who had been killed and praying for them to “be in a peaceful place.” The survivors also prayed for divine forces to help them obtain justice at the tribunal. Survivors and staff knelt down in front of the monks and offered three burning incense sticks symbolizing three generations: parents, children, and themselves. They also offered flowers and prayed. The *Achar* led the audience in chanting with some participants using the sacred language *Pali*, and others in their own Khmer language, while some people remained silent, keeping their hands in a *sampheas*, that is, held together in front of their heart. The monks joined the chanting in the sacred languages of *Pali* and *Sanskrit*, and sang about life and death. They sprinkled holy water on the audience in a ritual of purification and a blessing. The audience offered money to the monks on a dish, and two survivors stood up and spoke.

Following what had been a highly emotional experience at the “Killing Fields,” the participants arrived at the pagoda and gradually quieted down in the calm and soothing atmosphere. Survivors and TPO staff started by lighting incense sticks and praying to the Buddha statue at the pagoda. The *Achar* welcomed everyone, and the TPO director delivered a speech to honour the survivors ending with prayer.

One by one, the 16 testimonies, which had been summarized due to time restraints, were read out loud in front of the statue of Buddha by the counselors while other counselors stood by each survivor and showed support by touching his or her arm or shoulder. The counselors handed the testimony document to a monk, symbolically purifying the document. The survivor knelt in front of the monk and greeted him with a *sampheas* while the monk tied an orange thread around his or her right wrist. The orange thread is believed to have the magical powers to protect against and expel evil spirits. The monk offered the testimony document to the survivor and said words of blessing in *Pali*. Afterwards, the *Achar* held a common blessing of the whole audience, and the ceremony ended with everyone moving towards the monks who began chanting and sprinkling holy water and white jasmine flowers (symbols of purification) over the audience. Later, everyone shared a meal in a local restaurant.

At a group interview conducted just after the ceremony, sentiments by survivors included the following: “I feel relieved – I could express my feelings in the document and at the ceremony.” “I feel proud and excited about the ceremony.” “I received a gift: My painful story. I can show it to my children.” “I am happy because I received a document with my story.” “I feel good and relieved. I can laugh for the first time.” “My wound was opened and closed.”

At a group interview held the next day for staff members, they made comments such as: “Ceremonies like this could also be used as a means of community healing.” “The ceremony felt culturally appropriate: Everyone knew the meaning of the different phases.” “Next time, we could also ask the monks to allow the survivors ‘to give their suffering’ to the monks.” “I felt close to the victims. We felt close in ritual community.” “It helped me empathize with the survivor to read her story in the pagoda in the first person.”

### The Philippines

A workshop on testimonial therapy was held in the Philippines in April 2010 for the human rights organization Balay Rehabilitation Centre and some of its partner organizations. Twenty psychosocial practitioners attended the workshop including psychologists, human rights activists, and social workers. The trainees took testimonies from 13 survivors who had already been associated with Balay for some time. As part of the progressive development of the testimonial method during each workshop, the trainer tried, in cooperation with participants, to develop testimonial narratives, which would emphasize the strength and spirituality of the survivor. This was in response to challenges experienced in previous workshops regarding ways to develop healing narratives that more fully expressed sensations, images, and feelings in the course of dialogue between survivor and interviewer.

Due to the difference in religious beliefs and political ideology, three testimony ceremonies were held at the end of the workshop: one for the Catholic survivors, one for the Muslim survivors, and one for the political, mostly communist, ex-detainees. The workshop trainer took part in all of these ceremonies as a participant observer. After the workshop, Balay and one of its partner organizations conducted two additional ceremonies: One in honour of 10 former political prisoners who had been released as part of the peace process between their political group and the government. The other in honour of 15 civilians who survived atrocities committed by a terrorist organization.

The following describes the ceremony held for “Muslim brothers and sisters,” as the trainees called the survivors.

#### A Muslim ceremony

The ceremony was held in honour of four Muslim survivors of military torture and one female survivor whose husband was in prison. They had been suspected of belonging to rebel groups from the southern island of Mindanao that have been fighting for decades for a separate Islamic state. Since it was not possible to find a mosque near the training venue for the ceremony, the trainees decided to hold the ceremony in the conference room of the hotel where the workshop was conducted. The trainees decorated the room with flowers to convert the room into a “sacred space.” They also hung banners on the wall, the largest one bearing the words: “*Journeying with faith towards freedom: A testimony of life.*” The trainees had written the text themselves, and this banner seemed to encapsulate the central theme of the workshop. The key individuals conducting the ceremony were three imams and the Balay director. The trainees stressed that it was a great honour for Muslims when imams grace an occasion as they give the event a higher purpose and religious significance. Also present were selected family members of the survivors, the workshop trainer, and the counsellors who had taken the survivors’ testimonies. The audience consisted of family, friends, and staff members of the different organizations participating in the workshop.

The ceremony started with a *Kanduli*, a Muslim ritual meal usually performed at celebrations. The imams sat down with the survivors on the floor around the food in front of the audience. The meal began with a *duah*, or prayer, by an imam, after which the food was shared between the imams and the survivors.

The Balay director gave a speech honouring the survivors, after which each of the counsellors who had taken survivors’ testimonies read them out to the audience. The imams delivered the documents to the survivors, accompanying this with a prayer. Families and staff gave “messages of love, support and solidarity” to the survivors. One daughter made a tribute speech to her mother, then gave her flowers and hugged her. At the end of the speech both mother and daughter were crying as well as most of the audience. Children of the political detainees performed a Muslim dance. This was followed by a lunch in which food remaining from the sacred *Kanduli* meal was served and shared by the whole audience, reinforcing feelings of community and partaking in the blessings of the imams.

In a group interview immediately after the ceremony, survivors expressed thoughts such as: “I was happy to be reunited with my family who took part in the ceremony.” “I feel good that this happened to me.” “I feel liberated by being able to express the feelings that I have kept inside for many years.” “It feels good to see that many people understand me.” “It was very nice with prayers because prayers can do miracles. It felt very good to have the imams there so they could hear our stories. Maybe they can tell our stories to other members of the community.”

In a group interview with the staff after the ceremony, sentiments included the following: “Reading the testimony and placing oneself in the situation of the survivor is very helpful, I could better empathize with the survivor.” “The ceremony symbolized the trust of the survivors in us.” “I felt that our goals had been accomplished.” “My first impression of the Muslim people as terrifying was changed.” “Love was generously shared.” “I felt very overwhelmed because the survivor had doubts about reading the testimony in public.” “The presence of the family was very valuable.”

## Final remarks

The ceremonies that we developed during this experimental process showed that it is doable to integrate spirituality in testimonial therapy for Asian torture survivors. Once the workshop project was over, all NGOs continued to organize ceremonies in one form or another. The workshop trainer was a participant observer of nearly a third, that is, 14 of the 43 testimony ceremonies conducted in India, Sri Lanka, Cambodia, and the Philippines during the action research. The practical and logistical challenges of organizing a testimony ceremony required careful planning and were quite demanding for some organizations which had to strengthen their capacity in this respect. The idea of conducting an honour ceremony in connection with the testimonial process seemed to be highly acceptable to all Asian partners, as demonstrated by their enthusiastic response to the workshop trainer’s proposal and the feedback from survivors and staff after the ceremonies. On the whole, it seems to be a desirable brief therapeutic method. However, the workshop trainer also observed potential problems in connection with public exposure of the survivors, and especially women, in a ceremony. This involved ethical issues, which required careful consideration and planning. The staff had to find answers to this challenge and devise ways to ensure that the survivors really wished to have their story known by “the world” and understood their right to decline participation in a public ceremony after their testimony had been taken. Some organizations developed methods for honouring people such as rape survivors without exposing them in a potentially undesirable way.

## Discussion

The action research process, which has been analysed and reflected upon in this study, has explored the therapeutic implications of including cultural and religious ceremonies within testimonial therapy for torture survivors in South and Southeast Asia. The NGOs participating in the research process added new elements to the testimony method, including honour meetings in which the survivor’s testimony document was handed over in a forum locally regarded as a “ceremony.” This practice is in marked contrast with previous uses of the testimony method in other regions of the world in which the testimony document is given to the survivor with no attached formality or ceremony. In many cultures in South and Southeast Asia, a person is seen as a permeable being who is constituted through transactions in which the giving and receiving of material and nonmaterial elements occur (Marriott, 1976). Therefore, ceremonies become important occasions, as they seem to conform to the local meaning of “person.”

The research process, thus, led to a modification and reconceptualization of the testimonial method by including ceremonies performed in symbolic locations in which embodied spirituality is employed in various forms, such as songs, dances, religious purification rituals, meditation, the sharing of meals as well as public expression of love and compassion. The narratives that were read during the testimony ceremonies appeared to promote community support and symbolic reparations (Hamber, 2009) and facilitated self-awareness and spirituality (Fogel, 2009). The testimony ceremonies also seemed to function as *rites of passage* (van Gennep, 1960), marking a transition from the role of victim (Wilson, 2004) to an empowered survivor (Wilson, 2007) who can support others. These new types of ceremonial interactions transform the form and content of the testimonial therapy to include broader healing activities: the organization of pilgrimages to historical sites in which serious human rights crimes have been committed, and the denunciation of human rights violations.

From a strictly therapeutic perspective, the process of giving testimony may create a kind of forum in which there is a possibility for both an individual and collective interoceptive process. This can facilitate learning to tolerate feelings and sensations related to the traumatic memories (van der Kolk, 2006), and restructuring the traumatic memories by linking them to new and positive sensations (Hinton, Howes, & Kirmayer, 2008). The testimony ceremonies often had a high degree of emotional intensity and engaged the collective body within a palpable sense of *communitas* (Turner, 1969). However, more in-depth studies of different kinds of ceremonies and their impact on survivors are needed, as well as more studies on how ceremonial elements of the testimonial therapy could be applied in other geographic and cultural contexts. Cultures are no longer strictly local, but grow together and develop into novel hybrids, which may increase our capacity to heal the wounds caused by human rights violations.

The Asian testimonial therapy model with its emphasis on ceremonial practices described here, is not readily transferrable to a Western medical setting although elements of it might be useful in the rehabilitation of refugee torture survivors in Western countries. More research is needed to clarify how these insights can be used in other cultural and sociopolitical contexts. The results from our action research also suggest a need to further study the potential benefit of including mindfulness, an embodied approach facilitating interoception, and other spiritual practices into trauma treatment. The inclusion of measures of clinical outcome is needed to corroborate and advance our understanding of the findings of this study.

## Notes

1 Peoples’ Vigilance Committee for Human Rights, Varanasi, India; Janasansadya, Panadura, Sri Lanka; Home for Victims of Torture, Kandy, Sri Lanka; Transcultural Psychosocial Organisation – Cambodia, Phnom Penh, Cambodia; Balay Rehabilitation Center, Manila, Philippines.

2 21st Annual International Trauma Conference of the Trauma Centre in Boston, USA 20–22 May, 2010.

3 For a more detailed presentation of the method, see Perera et al. (2008).

4 Since the mid-1970s, discontent among the Tamil minority population in the northern and eastern parts of the country had given rise to the Liberation Tigers of Tamil Eelam (LTTE) movement and demands for a separate state. This resulted in a civil war that was fought with brutal intensity and claimed the life of more than 70,000 people. Most of the victims were civilian men, women, and children caught in-between the fighting. Emergency regulations and the Prevention of Terrorism Act allowed for the arrest and detention of not only Tamils but also any others even remotely suspected of supporting the LTTE along with anyone who questioned or opposed the military or the state. They were often detained for years on end and subjected to the most horrific forms of torture and cruel treatment. Sri Lanka has been a state party to the UN Convention Against Torture since 1994. Under domestic legislation, that is, the Convention Against Torture Act No 22 of 1994, any state official found guilty of torture, cruel, inhuman, or degrading treatment may be fined and sentenced to up to seven years of imprisonment. However, since investigations and prosecutions are conducted by the state itself, the accused continue to hold their official positions whilst being tried. Victims and witnesses are freely intimidated, threatened, attacked, and even killed. Charges are poorly framed and cases drag on for five or six years, and most of the accused are acquitted (Perera et al., 2008).

5 A person could file a complaint and become a civil party to the trial if he or she was recognized by the five judges. Civil parties had full procedural rights, that is, had access to the full case file and had the rights to make claims for reparations, which regular complainants and witnesses did not have. The tribunal, which is a national court supported by the UN, began its first trial in February 2009. Due to the great number of applicants, this procedure has been changed for the second case, which started in 2011. About 4000 people have been recognized as civil parties in Case 002.

6 A verdict was rendered on July 26, 2010 and appealed by both prosecutors and defence. On February 3, 2012 the Supreme Court Chamber sentenced Duch to life imprisonment.
